# Endovascular Treatment for Acute Ischemic Stroke in China: a study protocol for a prospective, national, multi-center, registry study

**DOI:** 10.3389/fneur.2023.1171718

**Published:** 2023-08-10

**Authors:** Liang Liu, Thanh N. Nguyen, Hui-Sheng Chen

**Affiliations:** ^1^Department of Neurology, General Hospital of Northern Theatre Command, Shenyang, China; ^2^Department of Neurology, Boston Medical Center, Boston, MA, United States; ^3^Department of Radiology, Boston Medical Center, Boston, MA, United States

**Keywords:** acute ischemic stroke, endovascular treatment, large vessel occlusions, protocol, registry study

## Abstract

**Background:**

Endovascular treatment (EVT) is the standard treatment for acute ischemic stroke (AIS) patients with large vessel occlusion (LVO).

**Aims:**

Endovascular Treatment for Acute Ischemic Stroke in China (DETECT2-China) aims to evaluate real-world outcomes and safety of EVT for LVO-AIS patients in China.

**Design:**

DETECT2-China is a prospective, national, multi-center study registered in ClinicalTrials.gov (NCT05092139). This study plans to enroll a total of 3,000 consecutive patients who received EVT for LVO. All eligible patients are adults aged 18 years or older with acute LVO who received EVT and standard medical treatment according to the Chinese stroke guidelines and local practice.

**Outcome:**

The primary outcome is functional independence (modified Rankin Scale score, mRS ≤ 2) at 90 days. The secondary outcomes include (1) the proportion of patients with mRS scores of 0–1 at 90 days, (2) distribution of mRS at 90 days, (3) changes in National Institutes of Health stroke scale (NIHSS) at 24 h, 48 h, and 12 days or discharge (whichever is earlier), (4) the proportion of symptomatic intracranial hemorrhage (sICH) within 48 h, and (5) the proportion of death within 7 days.

**Discussion:**

The DETECT2-China will provide real-world data about the effectiveness and safety of EVT for AIS-LVO patients in China.

## Introduction and rationale

Acute ischemic stroke (AIS) accounts for 60–80% of all stroke cases in China (1). Intravenous thrombolysis has been demonstrated to be associated with favorable functional outcomes for patients with AIS presenting within 4.5 h and is considered Class I evidence in national guidelines (2, 3). However, the efficacy of intravenous thrombolysis alone for patients with acute large vessel occlusion (LVO) is limited, and endovascular treatment (EVT) is often needed (4). Several studies have shown that EVT can achieve a high rate of recanalization and improve the outcome in LVO-AIS patients (4–9), which was further confirmed by the MR CLEAN REGISTRY and SVIN registry in routine clinical practice (10, 11). However, most of these studies were conducted in North America, Europe, and Australia. With the development of stroke centers in recent years, EVT has been performed in many Chinese hospitals. Given the restriction of EVT by equipment, cost, and experienced physicians, due to a different healthcare system in China, and due to the high prevalence of intracranial atherosclerosis (ICAS) in the Chinese AIS population (12), a multi-center, real-world registry study is needed to determine the real-world outcomes and safety of EVT for LVO-AIS patients in China, including imaging evaluation strategies and patient post-procedural management. In this context, this prospective, national, multi-center, real-world registry study is performed to evaluate the effectiveness and safety of the real-world practice of EVT in Chinese patients with LVO-AIS.

## Methods

### Design

Endovascular Treatment for Acute Ischemic Stroke in China (DETECT2-China) is a prospective, national, multi-center, registry study in China and intends to include 30 comprehensive stroke centers (Supplementary material) between January 2022 and December 2024 to characterize the real-world clinical outcomes and safety of EVT for patients with LVO-AIS in China.

### Study population

In the DETECT2-China prospective registry, eligible participants are LVO-AIS patients who received EVT. The detailed inclusion and exclusion criteria are shown in Table 1. To avoid selection bias, all participating centers are obliged to enter consecutive patients undergoing EVT in this study.

**Table 1:** Inclusion and exclusion criteria.

**Inclusion criteria**
•Age ≥ 18 years
•Ischemic stroke confirmed by head CT or MRI and vessel occlusion confirmed by CTA, MRA, or DSA
•Patients receiving endovascular treatment
•First ever stroke or pre-stroke mRS ≤ 2
•Signed informed consent by a patient or their legally authorized representative.
**Exclusion criteria**
•The presence of intracranial hemorrhage at the baseline neuroimaging
•Pre-stroke mRS ≥ 3
•Other serious illness that would confound the clinical outcome at 90 days
•Unavailable neuroimaging data
•Other unsuitable conditions judged by the investigator.

CT, computed tomography; MRI, magnetic resonance imaging; mRS, modified Rankin Scale.

### Standard protocol approval, registration, and patient consent

The protocol of DETECT2-China and data collection have or will have been approved by the Ethics Committee of the General Hospital of Northern Theater Command and all participating sites. All patients are enrolled in the study voluntarily, and patients or their legally authorized representatives sign informed consent. The DETECT2-China trial is registered on www.clinicaltrial.gov (NCT05092139).

### Baseline characteristics

Baseline patient demographics including age, sex, pre-stroke mRS, vascular risk factors, and smoking use will be recorded. Stroke benchmark metrics such as baseline NIHSS, time from last known well, arrival to the hospital, neuroimaging modality and time, and use and time of administration of IVT will be collected. The etiology of the stroke will also be recorded according to the TOAST criteria.

### Intervention

Intravenous thrombolysis with alteplase, tenecteplase, or urokinase can be used before and/or during EVT according to local or national guidelines. Femoral artery or radial artery puncture with local anesthesia is used for EVT. The choice of the anesthetic strategy (conscious sedation or general anesthesia) will be determined by the anesthesiology team and the neurointerventionalist according to a case-by-case basis. EVT will be carried out according to the usual practice of each center, including EVT with stent retrievers, thromboaspiration, balloon angioplasty, stenting, or a combination of these approaches. Intra-arterial thrombolysis with alteplase, tenecteplase, or urokinase and antithrombotic treatment with tirofiban, aspirin, clopidogrel, cilostazol, or ticagrelor are permitted in this registry at the operator's discretion. Systolic blood pressure and diastolic blood pressure will be recorded. Digital subtraction angiography (DSA) and a cone beam CT (or non-contrast CT) will be performed after EVT to evaluate recanalization and exclude intracranial hemorrhage. The number of passes to achieve successful recanalization as well as the use of adjunctive antithrombotics, if pertinent, will be recorded. Recanalization is graded according to the modified thrombolysis in cerebral infarction (mTICI) score, and successful recanalization is defined as having an mTICI grade of 2b-3.

### Outcomes

The primary outcome is the proportion of patients who achieve functional independence (modified Rankin Scale score, mRS ≤ 2) at 90 days. The minimum and maximum values of the modified Rankin Scale score are 0 and 6, respectively; a higher score means a worse outcome with 6 indicating death.

The secondary outcomes include the following: (1) the percentage of patients with excellent functional outcome, defined as an mRS score of 0 to 1 at 90 days, (2) distribution of mRS at 90 days, (3) changes in National Institutes of Health stroke scale (NIHSS) at 24 h, 48 h, and 12 days, (4) proportion of symptomatic intracranial hemorrhage (sICH) within 48 h, defined as an increase in 4 or more points on the NIHSS that is caused by intracranial hemorrhage (ECASS-III definition) (2), (5) proportion of death within 7 days, (6) procedural complications, including vessel dissection, contrast extravasation, embolization into a new territory, femoral access complications, and (7) discharge disposition.

Exploratory outcomes include the following: (1) the association between neuroimaging within 48 h and clinical outcome (neuroimaging includes brain CT, MRI, or DSA); (2) changes in serum biomarkers at 48 h, including matrix metalloproteinases, proinflammatory cytokines, brain-derived neurotrophic factor (BDNF), neurofilament light chain, serum complement C3, C-reactive protein, albumin, copeptin, D-dimer, and uric acid; (3) transcranial Doppler ultrasound data within 24 h, including blood velocity and pulsatility index, which will be collected at some centers; and (4) dynamic changes of cortical blood oxygen saturation by near-infrared spectroscopy within 24 h, which will be collected at some centers.

### Follow-up protocol

All included patients will have an in-person or telephone interview at the baseline, 24 ± 4 h, 48 ± 8 h, 7 ± 1 days, 12 ± 2 days or at discharge if earlier, and 90 ± 7 days post-EVT, respectively (Table 2). All data will be collected using electronic case report forms (eCRFs) and an online electronic data capture (EDC) system.

**Table 2:** Timing of all procedures in DETECT-2.

**Time point**	**Screening**	**24 ±4 h**	**48 ±8 h**	**7 ±1 days**	**12 ±2 days**	**90 ±7 days**
**Enrollment**						
Eligibility screen	x					
Informed consent	x					
**Interventions**						
Intravenous thrombolysis	x					
Endovascular treatment	x					
**Clinical assessment**						
Diagnosis	x					
Head CT/MRI	x	x	x			
Cortical oxygen monitoring^a^		x				
TCD monitoring^b^		x				
NIHSS assessment	x	x	x	x	x	
mRS assessment	x					x
Medical history	x					
Demographic characteristics	x					
Vital signs	x	x	x	x	x	x
mTICI score	x					
TOAST classification					x	
**Laboratory examination**						
Blood routine	x					
Urine routine	x					
Coagulation	x	x	x		x	
Serum biochemistry	x				x	
ECG	x				x	
24-h ECG monitor				x		
**Follow-up record**						
Concomitant medications		x	x	x	x	x
Adverse events		x	x	x	x	x
Recurrent stroke and other vascular events		x	x	x	x	x

CT, computed tomography; MRI, magnetic resonance imaging; TCD, transcranial Doppler ultrasound; NIHSS, National Institutes of Health Stroke Scale; mRS, modified Rankin Scale; mTICI, modified thrombolysis in cerebral infarction; TOAST, Trial of Org 10172 in Acute Stroke Treatment; ECG, electrocardiogram.

^a^Continuous cortical oxygen monitoring was performed immediately after the endovascular treatment (monitoring 24 h).

^b^TCD monitoring was performed immediately, 6, 12, and 24 h after endovascular therapy.

### Data management, monitoring, and quality control

All patient data and case report forms will be entered in MedSci (http://DETECT2-China.medsci.cn). The data will be downloaded from MedSci with a dedicated person for statistical analysis. A third-party contract research organization (CRO) and the sponsor will audit the sites regularly to ensure adherence to study documentation, reporting procedures, and study protocol. The registry steering committee is comprised of external scientific advisors and will organize teleconference or in-person meetings to give recommendations about the study. Neuroradiologic assessment will be conducted in a core laboratory. Neuroimaging associated with clinical events will be collected centrally and interpreted by two independent neuroradiologists. An independent Endpoint Events Assessment Committee will adjudicate controversial clinical and imaging outcomes. The trial is initiated and supported by the Cerebrovascular Disease Collaboration and Innovation Alliance (CDCIA) of Liaoning.

### Sample size determination

According to a previous report (9), the proportion of functional independence at 90 days (mRS of 0–2) was 46% after EVT within 7 h of onset. After a two-sided test was performed with a power of 80% and an α set at 0.05, the calculated sample size is 1,565 cases. Considering a loss to follow-up rate of 25%, the sample size was therefore readjusted to 1,800 cases. Considering that patients with anterior circulation occlusion within 7 h of onset account for ~60% of the enrolled population, this registry has been designed to include 3,000 patients.

### Statistical analysis

Statistical analyses will be performed based on all enrolled participants or their legally authorized representatives who signed and did not withdraw informed consent. All data will be analyzed with SPSS 23.0 Software. The mean ± standard deviation (SD) will be used if continuous data are normally distributed, and the median and interquartile range (IQR) will be used if continuous data are non-normally distributed. Categorical data are expressed as a number (percentage). Differences between the primary endpoint and secondary endpoints such as mRS (0–1) at 90 days, the proportion of symptomatic intracranial hemorrhage at 48 h, the proportion of intraparenchymal hemorrhage at 48 h, and the occurrence of all-cause mortality at 7 days will be compared using binary logistic regression. The analysis will be adjusted for confounders, including age, sex, baseline NIHSS, pre-stroke mRS, prior stroke, baseline ASPECTS, site of arterial occlusion, transfer of patient, time from last known well, and IVT use (for EVT patients). The 90-day mRS score will be compared using ordinal logistic regression. Change in the NIHSS score will be compared using a general linear model. Time to events of stroke recurrence and other vascular events will be compared using Cox regression. Statistical tests are considered significant when the two-sided *P*-value is < 0.05.

The outcomes in DETECT2-China will be further analyzed by the following subgroups: (1) onset of symptoms to recanalization time (<7 vs. ≥7 h), (2) age (<65 vs. ≥65), (3) NIHSS score on admission (6–16 vs. ≥17), (4) baseline ASPECTS (<6 vs. ≥6), (5) thrombectomy technique (stent retriever vs. contact aspiration or other techniques), (6) anterior circulation vs. posterior circulation, (7) mean systolic blood pressure during the intervention (≤ 120 vs. >120 mmHg), (8) mechanical thrombectomy alone vs. bridging thrombectomy, (9) mothership vs. drip and ship models, (10) stroke etiology (ICAD vs. cardioembolic vs. others), and (11) use of general anesthesia vs. conscious sedation.

## Discussion

The DETECT2-China is a prospective, multi-center registry, aiming to evaluate the clinical outcomes and safety of EVT in Chinese LVO-AIS patients.

This trial has two distinct characteristics. First, this is a real-world registry in China. Differences in body mass, comorbidities, and etiology of AIS patients between different ethnicities are of interest to study as the five early LVO randomized clinical trials were performed in America, Europe, and Australia (4, 6–9). The subjects included in randomized clinical trials are more homogeneous in baseline characteristics, while the inclusion and exclusion criteria of DETECT2-China are not restrictive given the design of the real-world registry (13). Thus, DETECT2-China is expected to provide real-world data covering a comprehensive group of patients, and this could be helpful to understand some of the main controversies in EVT, for example, the benefit of EVT for AIS with large infarct core, very low or very high NIHSS due to LVO, medium-sized vessel occlusions, posterior circulation strokes, isolated cervical carotid occlusions, extreme ages, extended time window without advanced neuroimaging selection, etc. In addition, this registry will provide valuable data about different EVT modality treatment paradigms such as mothership vs. drip and ship. Second, this registry will enroll all patients who received EVT such as intra-arterial thrombolysis, stent retrievers, thromboaspiration, balloon angioplasty, stenting, or various combinations of these approaches. The design is consistent with clinical practice. Collectively, DETECT2-China should reveal comprehensive data about EVT for Chinese LVO-AIS patients in a real-world setting.

## Limitations

As this is a prospective registry that enrolls only patients or their legally authorized representatives who provide consent, we may not be able to capture patients who do not or are unable to provide informed consent. Moreover, since this is a real-world registry study, other limitations are inevitable, including selection bias, a lack of standardization of techniques and treatment protocols, variability in operator experience, a heterogeneous patient population, and a lack of a control group.

## Ethics statement

The studies involving human participants were reviewed and approved by the Ethics Committee of General Hospital of Northern Theater Command. The patients/participants provided their written informed consent to participate in this study.

## Author contributions

LL and H-SC wrote the first draft of the manuscript. TN critically revised the manuscript. H-SC designed the study and critically revised the manuscript. All authors have carefully read and approved the article.
