# Supplementary material for: Endovascular Treatment for Acute Ischemic Stroke in China: a study protocol for a prospective, national, multi-center, registry study
Source: Front Neurol. 2023 Aug 10;14:1171718. doi: 10.3389/fneur.2023.1171718 (PMC10447973; doi:10.3389/fneur.2023.1171718)
Supplement: Supplementary file 1 [file Table_1.DOCX]

# RECRUITMENT BY SITE

| **Number** | **Participating centers** |
| --- | --- |
| 1 | General Hospital of Northern Theater Command, Shenyang, China |
| 2 | General Hospital of Fushun Mining Bureau of Liaoning Health Industry Group, Fushun, China |
| 3 | General Hospital of Fuxin Mining Bureau of Liaoning Health Industry Group, Fuxin, China |
| 4 | The Second Affiliated Hospital of Harbin Medical University, Harbin, China |
| 5 | Panjin Central Hospital, Panjin, China |
| 6 | Huludao Central Hospital, Huludao, China |
| 7 | Beipiao Central Hospital, Beipiao, China |
| 8 | Shengli Oilfield Central Hospital, Dongying, China |
| 9 | The First Hospital of Jilin University, Changchun, China |
| 10 | Dalian Municipal Central Hospital, Dalian, China |
| 11 | Anshan Changda Hospital, Anshan, China |
| 12 | Liaocheng Brain Hospital, Liaocheng, China |
| 13 | Yingkou Central Hospital, Yingkou, China |
| 14 | The First Affiliated Hospital of Harbin Medical University, Harbin, China |
| 15 | Mianyang Central Hospital, Mianyang, China |
| 16 | Tiemei General Hospital of Liaoning Health Industry Group, Diaobingshan, China |
| 17 | Tongliao City Hospital, Tongliao, China |
| 18 | Nanyang Central Hospital, Nanyang, China |
| 19 | Tianjin Huanhu Hospital, Tianjin, China |
| 20 | The First Affiliated Hospital of Dalian Medical University, Dalian, China |
| 21 | Fuxin Second Hospital, Fuxin, China |
| 22 | Jiamusi Central Hospital, Jiamusi, China |
| 23 | Dandong Central Hospital, Dandong, China |
| 24 | Yantai Yuhuangding Hospital, Yantai, China |
| 25 | The Second Affiliated Hospital of Lanzhou University, Lanzhou, China |
| 26 | Gansu Province Central Hospital, Lanzhou, China |
| 27 | Liaoyang Second Hospital, Liaoyang, China |
| 28 | Benxi Central Hospital, Benxi, China |
| 29 | Linfen Central Hospital, Linfen, China |
| 30 | Nanshi Hospital of Nanyang, Nanyang, China |
